# KITLG is a novel target of *miR-34c* that is associated with the inhibition of growth and invasion in colorectal cancer cells

**DOI:** 10.1111/jcmm.12368

**Published:** 2014-09-12

**Authors:** Shu Yang, Wen-shuai Li, Fang Dong, Hai-mei Sun, Bo Wu, Jun Tan, Wan-jing Zou, De-shan Zhou

**Affiliations:** 1 Department of Histology and Embryology, School of Basic Medical Sciences, Capital Medical UniversityBeijing, China

**Keywords:** colorectal cancer cell, KITLG, *miR-34c*, tumour suppressor

## Abstract

*MiR-34c* is considered a potent tumour suppressor because of its negative regulation of multiple target mRNAs that are critically associated with tumorigenesis and metastasis. In the present study, we demonstrated a novel target of *miR-34c*, KITLG, which has been implicated in colorectal cancer (CRC). First, we found a significant negative relationship between *miR-34c* and *KITLG* mRNA expression levels in CRC cell lines, including HT-29, HCT-116, SW480 and SW620 CRC cell lines. *In silico* analysis predicted putative binding sites for *miR-34c* in the 3′ untranslated region (3′UTR) of *KITLG* mRNA. A dual-luciferase reporter assay further confirmed that *KITLG* is a direct target of *miR-34c*. Then, the cell lines were infected with lentiviruses expressing *miR-34c* or a *miR-34c* specific inhibitor. Restoration of *miR-34c* dramatically reduced the expression of *KITLG* mRNA and protein, while silencing of endogenous *miR-34c* increased the expression of KITLG protein. The *miR-34c*-mediated down-regulation of KITLG was associated with the suppression on proliferation, cellular transformation, migration and invasion of CRC cells, as well as the promotion on apoptosis. Knockdown of KITLG by its specific siRNA confirmed a critical role of KITLG down-regulation for the tumour-suppressive effects of *miR-34c* in CRC cells. In conclusion, our results demonstrated that *miR-34c* might interfere with KITLG-related CRC and could be a novel molecular target for CRC patients.

## Introduction

The 20–24 nucleotide microRNAs post-transcriptionally regulate the expression of their target genes *via* either translational repression or mRNA degradation by binding to the complementary seed sites within the 3′ untranslated region (3′UTR) of the target mRNA. It is widely believed that microRNAs modulate diverse biological processes, and aberrant expression of microRNAs has been implicated in multiple pathological processes, including tumorigenesis and metastasis, as these genes typically serve as either oncogenes or tumour suppressor genes, depending on their targets 1–5. Recent works have shown that Overexpression of *miR-34c* induces apoptosis and inhibits cell proliferation and invasion in a variety of tumours 6–10. Therefore, *miR-34c* is considered a potent tumour suppressor. The tumour-suppressive role of *miR-34c* can be attributed to the down-regulation of its target genes that are critically associated with cell apoptosis, proliferation and invasion. For example, *miR-34c* negatively regulates the oncogenes *E2F3* and *BCL-2* and subsequently suppresses proliferation and stimulates apoptosis in prostate cancer cells 8. *MiR-34c* was also found to silence *BMF* and *c-MYC*, which contribute to the cell's resistance to paclitaxel-induced apoptosis 10. Ectopic expression of *miR-34c* inhibits the epithelial-mesenchymal transition (EMT) and suppresses migration of breast tumour-initiating cells through the silencing of *NOTCH4* 9. Additionally, a few publications have shown that *miR-34c* is down-regulated in colorectal cancer (CRC) specimens and plays a tumour-suppressive role in CRC by targeting *SNAIL1*, a transcriptional repressor linked to the EMT programme and tissue-invasive activity, and some components of the canonical Wnt signalling cascades, which control cancer stem cell generation and cancer progression 11–14.

Colorectal cancer is the 3rd most common gastrointestinal cancer and one of the leading causes of death worldwide. With emerging evidence supporting the tumour-suppressive role of *miR-34c*,*miR-34c* down-regulation could be one of the causes and novel therapeutic strategies for CRC. However, because the neoplastic growth and invasion of CRC are a result of a network of multiple signalling molecules, efforts are being made to figure out how *miR-34c* is involved in CRC suppression. KITLG, also known as stem cell factor, steel factor and mast cell growth factor, has multiple biological functions during the development of mice, rats and humans by triggering its receptor tyrosine kinase, c-KIT 15–17. Interest in KITLG has been bolstered by work showing that aberrant expression of KITLG has been implicated in the development of several cancers. For instance, elevated KITLG expression contributes to the carcinogenesis of uveal melanoma 18, neurofibromatosis type 1 19, gliomagenesis 20, breast cancer 21 and non-small-cell lung cancer 22, presumably because of the c-KIT/KITLG autocrine/paracrine stimulation-loop mechanism 23,24. Moreover, Bellone *et al*. 25–27 reported that CRC tissues over-express KITLG, which is required for cancer cell growth, migration and invasion. However, the regulation of KITLG expression in CRC still remains largely unexplored.

Therefore, in the present study, we investigated the interaction between *miR-34c* and KITLG in CRC cell lines, and demonstrated that KITLG is a direct target of *miR-34c* and mediates the role that *miR-34c* plays in proliferation, migration and invasion in CRC cells. Our results might be helpful in improving the current understanding of CRC development, as well as improving the diagnosis and treatment of CRC.

## Materials and methods

### Cell culture

The human CRC cell lines HT-29, HCT-116, SW480 and SW620, as well as the African green monkey kidney fibroblast COS-7 cell line, were purchased from ATCC. The HT-29 and HCT-116 cell lines were cultured in DMEM (Gibco, Carlsbad, CA, USA); the SW480 and SW620 cells were grown in RPMI-1640 (Gibco); the COS-7 cells were grown in Opti-MEM (Gibco). All mediums were supplemented with 10% foetal bovine serum (Invitrogen, Carlsbad, CA, USA) and 1% penicillin/streptomycin (P/S, Gibco). The cells were grown at 37°C in the presence of 5% CO_2_.

### Immunofluorescence

The cells were fixed with 4% paraformaldehyde for 30 min. Non-specific binding was blocked by incubation with 1% bovine serum albumin (Sigma-Aldrich, St. Louis, MO, USA) for 1 hr. The cells were incubated with the mouse anti-KITLG (1:200; Santa Cruz, Dallas, TX, USA) or rabbit anti-c-KIT (1:200; Cell Signaling Technology, Beverly, MA, USA) primary antibody overnight at 4°C, followed by incubation with the corresponding secondary antibodies for 1 hr at 25°C. The cells were mounted by using DAPI mounting medium (Zhongshan Jinqiao Biotechnology, Beijing, China) and were observed with a fluorescence microscope (Nikon, 80i, Tokyo, Japan). The negative control cells were incubated with an isotype control antibody or without a primary antibody.

### Lentiviral vector construction and infection

The full length *pre-miR-34c* was chemically synthesized by GeneChem and was introduced into the GV217 lentiviral vector (GeneChem, Shanghai, China, Fig. S1) in the unique EcoRI site to construct a lentivirus encoding *miR-34c* (lenti-*miR-34c*); this construct was confirmed by using nucleotide sequencing. The specific inhibitor of *miR-34c* was constructed by cloning the complementary nucleotides of *miR-34c* into the GV280 lentiviral vector (GeneChem, Fig. S1) between the AgeI and EcoRI sites. The cells were seeded in a 6-well plate at a density of 5 × 10^4^ cells/well and were infected with lenti-*miR-34c* or its inhibitor when the cells reached 30% confluence. Three days after infection, the efficiency of infection was evaluated by observing the EGFP expression by using a fluorescence microscope (Nikon, 80i).

### RNA extraction and Real-time PCR

Total RNA was extracted from the cultured cells by using the TRIzol reagent (Invitrogen), and microRNA was extracted by using the miRNApure Mini Kit (CWBiotech, Beijing, China), according to the manufacturers’ instructions.

For mRNA, the reverse transcription reactions were performed with the Super cDNA First-Strand Synthesis Kit (CWBiotech). Real-time PCR was performed in an ABI 7500 real-time PCR system (Applied Biosystems, Carlsbad, CA, USA) by using the Ultra SYBR Mixture with ROX (CWBiotech). The following primers were used: *KITLG* (Forward: CAGAGTCAGTGTCACAAAACCATT, Reverse: TTGGCCTTCCTATTACTGCTACTG); *c-KIT* (Forward: GATTATCCCAAGTCTGAGAATGAA; Reverse: CGTCAGAATTGGACACTAGGA); *GAPDH* (Forward: AGAAGGCTGGGGCTCATTTG, Reverse: AGGGGCCATCCACAGTCTTC). The reactions were incubated at 95°C for 10 min., followed by 40 cycles of 95°C for 15 sec. and 60°C for 1 min.

For microRNA, reverse transcription was performed with the Taqman microRNA RT Kit (Invitrogen) and the Taqman microRNA Assay with specific stem-loop primers (Assay ID 428 for *miR-34c* and 1973 for *RNU6B*; Invitrogen). Real-time PCR was performed with the Taqman Universal Master Mix II (Invitrogen) and the Taqman microRNA Assay (Invitrogen). The reactions were incubated at 95°C for 10 min., followed by 40 cycles of 95°C for 15 sec. and 60°C for 1 min.

All reverse transcription reactions included no-template controls, and all PCR reactions were run in triplicate. Relative gene expression was determined by using the comparative C_T_ (2^−ΔΔCt^) method.

### Western blot analysis

The harvested cells were suspended in cell lysis buffer. After 12% SDS-PAGE, the proteins were transferred onto a PVDF membrane (Millipore, Temecula, CA, USA) and blocked with Tris-buffered saline containing 0.05% Tween-20 (TBST) and 5% non-fat dry milk for 1 hr. The membrane was incubated with the rabbit anti-KITLG (1:500; Abcam, Cambridge, UK) or rabbit anti-c-KIT (1:1000; Cell Signaling Technology) primary antibodies at 4°C overnight. Then, the membrane was incubated with HRP-conjugated secondary goat anti-rabbit IgG (1:2000; Santa Cruz) for 1 hr. The proteins were detected by using ECL chemiluminescence. A mouse anti-GAPDH (1:1000; Applygen, Beijing, China) antibody was used as an internal control.

### Soft agar colony formation assay for anchorage-independent growth

The bottom of a 6-well plate was coated with 0.6% low melting agarose (Promega, Madison, WI, USA), which was then covered with 1.4 ml of 0.35% agarose containing 1000 cells either infected with *miR-34c* or its inhibitor. The 6-well plate was incubated for 14 days at 37°C with 5% CO_2_. Colonies were photographed by using an inverted phase contrast microscope (Leica DMI3000 B, Wetzlar, Germany). Ten visual fields were sampled with a systemic random sampling method, and the colonies were counted through stereological technique.

### Real-time monitoring of cellular proliferation, migration and invasion

We employed the ‘xCELLigence’ system (ACEA, Biosciences, San Diego, CA, USA), which consists of E-plates and the Real Time Cell Analyzer Dual Purpose (RTCA-DP) instrument, to monitor cell proliferation by measuring the cell index (CI), which is proportional to the number of cells 28. The cells were seeded in E-plates at a density of 8000 cells/well. The E-plates were then transferred to the RTCA-DP instrument for automated real-time monitoring at standard incubator conditions; CI measurements were collected every 15 min. for the following 96 hrs.

Cellular migration and invasion were also monitored by using the ‘xCELLigence’ system on CIM (Cell Invasion-and-Migration)-plates, instead of E-plates. The CIM-plates are made by integrating microelectronic sensors onto the underside of the microporous membrane of the upper chamber. Cells capable of migrating from the upper chamber through the microporous membrane into the bottom chamber will contact and adhere to the sensors, leading to an increase in the impedance signals detected by the sensors. The impedance increase is correlated with numbers of cells that have migrated to the underside of the membrane. Therefore, cell migration activity can be monitored with the impedance readouts 28. Our migration assays were performed by seeding cells in the upper chambers of the CIM-plates in serum-free medium at a density of 20,000 cells per well. The bottom chambers of the CIM-plates were filled with serum-containing medium to promote migration across the membranes towards the serum gradient. After seeding, the CIM-plates were transferred into the RTCA-DP instrument for real time read-outs during 48 hrs. Impedance or the CI was registered only from the cells that were capable of migrating through the membranes. Four independent experiments were performed. For the invasion assays, the protocol was identical to that for the migration assay, except that the upper chambers were loaded with 30 μl of a 10% Matrigel (BD Biosciences, Franklin Lakes, NJ, USA) solution to create a 3D biomatrix film in each well prior to cell seeding.

### Apoptosis analysis

Cell apoptosis was detected with the Alexa Fluor® 488 Annexin V/Dead Cell Apoptosis Kit with Alexa® Fluor 488 Annexin V and PI for Flow Cytometry (Invitrogen) according to the manufacturers’ instruction.

### Dual-luciferase reporter assay

The 3′UTR sequences of *KITLG* containing the predicted seed regions of *miR-34c* were chemically synthesized by GeneChem. The fragment was introduced into the GV306 luciferase reporter vector at the unique XbaI site, which is downstream of the *Firefly* luciferase stop codon and is followed by the *Renilla* luciferase sites (GeneChem, Fig. S1). The seed regions of *miR-34c* in the *KITLG* 3′UTR were mutated to construct GV306-*KITLG*-MUT. The GV262-*miR-34c* construct was created by introducing the *miR-34c* sequence into the GV262 vector at the XhoI/EcoRI sites (GeneChem, Fig. S1). A construct containing an unrelated microRNA was used as a negative control (GV262-miR-control).

The GV262-*miR-34c* construct was transiently transfected with GV306-*KITLG* or GV306-*KITLG*-MUT into COS-7 cells by using Lipofectamine 2000 (Invitrogen), according to the manufacturer's instructions. Twenty-four hours after transfection, the *Firefly* and *Renilla* luciferase activities were measured by using the Dual Luciferase Assay System (Promega) in a Modulus™ II Microplate Multimode Reader (Promega), according to the manufacturers’ protocols. The ratio of *Firefly*/*Renilla* activity was calculated.

### *KITLG*-specific small interfering RNA (siRNA) treatment

For specific gene knockdown on *KITLG* mRNA, siRNA targeting KITLG and negative control siRNA were purchased from Ribobio Co., Ltd (Guangzhou, China). siRNAs were transfected into HCT-116 cells by using riboFECT™ CP Transfection Kit (Ribobio) according to the manufacturer's instructions. Total RNA and total protein were prepared 48 hrs post-transfection and used for real-time PCR and western blot analysis, respectively. Furthermore, cellular proliferation, invasion and apoptosis after siRNA treatment were performed.

### Statistics

The results were presented as the mean ± SEM and analysed by using a Student's *t*-test or one-way anova with the SPSS 13.0 software (Chicago, IL, USA). Bivariate correlation was analysed by using the Spearman method with SPSS 13.0. A *P*-value of 0.05 or less was considered statistically significant.

## Results

### Expression of KITLG and c-KIT in CRC cell lines

**Figure 1:**
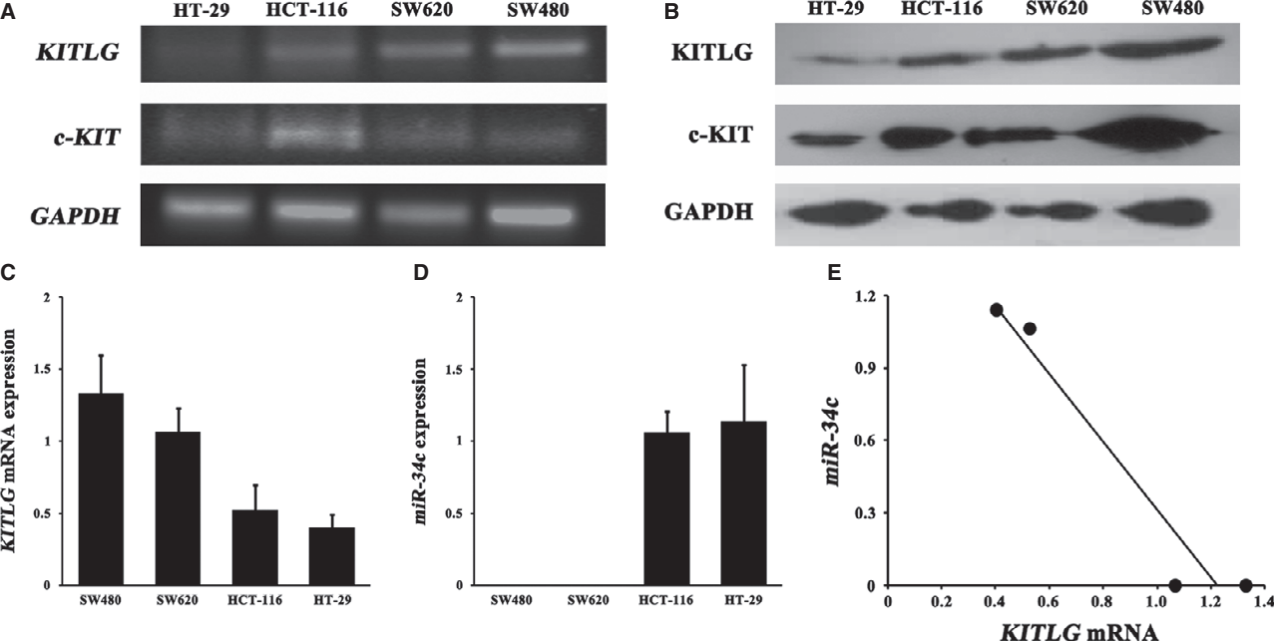
All of the HT-29, HCT-116, SW480 and SW620 cells co-express KITLG and c-KIT at both the mRNA (**A**) and protein levels (**B**). *KITLG* mRNA expressions (*n* = 4) in the 4 CRC cell lines were inversely correlated with the expression of *miR-34c* (*n* = 4; *P* < 0.05, **C**–**E**).

The autocrine/paracrine stimulation loop of c-KIT/KITLG signalling is one mechanism of malignant cells 25. Therefore, we first screened four CRC cell lines for the presence of the mRNA and protein of KITLG and its receptor c-KIT The mRNA and protein expressions of *KITLG* and *c-KIT* were determined in these CRC cell lines (Fig.1A and B).

### *miR-34c* is inversely correlated with *KITLG* mRNA expression

All CRC cells showed apparent but variable levels of *KITLG* mRNA expression (Fig.1C). Although *miR-34c* was not detectable in the SW480 and SW620 cells, it was expressed in the HCT-116 and HT-29 cells (Fig.1D). Notably, statistical analysis revealed a significant inverse correlation between the endogenous *KITLG* mRNA and *miR-34c* expression levels (*r*_s_ = −0.913, *P* < 0.05; Fig.1E).

### *KITLG* is a direct target of *miR-34c*

**Figure 2:**
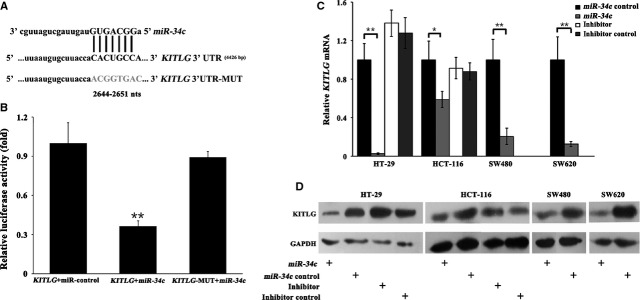
(**A**) Schematic diagram of the *KITLG* 3′UTR and the putative seed-matching sequences (2644-2651 nts in uppercase) that are complementary to the *miR-34c* sequences. The 8 mer seed-matching sequence was mutated (in grey) so that *miR-34c* could no longer bind to the *KITLG* 3′UTR. (**B**) COS-7 cells were transiently transfected with the luciferase reporter plasmid containing the *KITLG* 3′UTR and *miR-34c*. Luciferase activity was evaluated and normalized to the miR-control. The luciferase activity was significantly decreased in cells cotransfected with the *KITLG* 3′UTR and *miR-34c* (**P < 0.01), but not in cells cotransfected with the *KITLG* 3′UTR-MUT and *miR-34c*. After infection with the lentiviral-mediated *miR-34c*, the mRNA (**C**) and protein (**D**) levels of KITLG in the HT-29, HCT-116, SW480 and SW620 cells were significantly reduced compared to the lenti-control infected cells (*P < 0.05; **P < 0.01). On the contrary, infection with the *miR-34c* inhibitor reversed the decrease in KITLG protein in the HT-29 and HCT-116 cells; however, *KITLG* mRNA was not affected. The *miR-34c* inhibitor had no effect on KITLG protein expression in the SW480 and SW620 cells, which do not express *miR-34c*.

We performed *in silico* analysis with the TargetScanHuman, MiRanda and miRBase programmes to search for all putative targets of *miR-34c*, and a cross-checked list revealed hundreds of predicted targets. For the purposes of uncovering the relevant targets that would modulate the phenotypic effects in CRC, we then performed a Functional Annotation Clustering of the combined predicted targets by using the DAVID program (http://david.abcc.ncifcrf.gov/). The KEGG pathways that were enriched with potential *miR-34c* targets (enrichment score = 2.15) were cancer related (*P* = 0.015), and *KITLG* was one of the most likely targets within these pathways in cancer. We further predicted a potential *miR-34c* seed region at nt 2644-2651 of the *KITLG* 3′UTR (NM_000899). To determine whether *KITLG* is a *bona fide* target of *miR-34c*, we constructed a luciferase reporter plasmid containing the seed region of the *KITLG* 3′UTR that was complementary to *miR-34c* (Fig.2A). Additionally, we generated a mutant reporter in which the whole 8 nucleotides of the seed region in the *KITLG* 3′UTR were mutated (Fig.2A). Using a dual-luciferase reporter assay, transfection of the *KITLG* 3′UTR in combination with *miR-34c* significantly reduced the luciferase activity, whereas *miR-34c* had no influence on the luciferase activity of the mutated *KITLG* 3′UTR construct (*P* < 0.01, Fig.2B), suggesting that the inserted fragment from the *KITLG* 3′UTR (nt 2644-2651) is a direct target of *miR-34c*.

To assess the consequences of the up-or down-regulation of *miR-34c*, CRC cells were infected with *miR-34c* or its inhibitor, and the mRNA and protein levels of KITLG were analysed. Three days post-infection, the infection efficiency of *miR-34c* was determined by immunofluorescence staining and real-time PCR analysis and suggested that the lentiviral-mediated *miR-34c* constructs were efficiently transduced into the CRC cells; consequently, the expression level of *miR-34c* was significantly increased compared to the controls (*P* < 0.01), whereas the level of the unrelated *miR-214* remained unchanged (*P* > 0.05; Fig. S2). As expected, both the mRNA and protein levels of KITLG in the cells infected with *miR-34c* were significantly decreased (*P* < 0.05 or 0.01, Fig.2C and D). In contrast, the silencing of endogenous *miR-34c* by its specific inhibitor resulted in the increased expression of KITLG protein (*P* < 0.05, Fig.2D), but not *KITLG* mRNA, in the HCT-116 and HT-29 cells (*P* > 0.05, Fig.2C). Because *miR-34c* was undetectable in the SW480 and SW620 cells, we did not perform the *miR-34c* inhibitor infection in these two cell lines. These results indicated that *miR-34c* negatively regulates the expression of *KITLG* in CRC cells.

### *miR-34c*-mediated down-regulation of KITLG is associated with cellular proliferation and apoptosis

**Figure 3:**
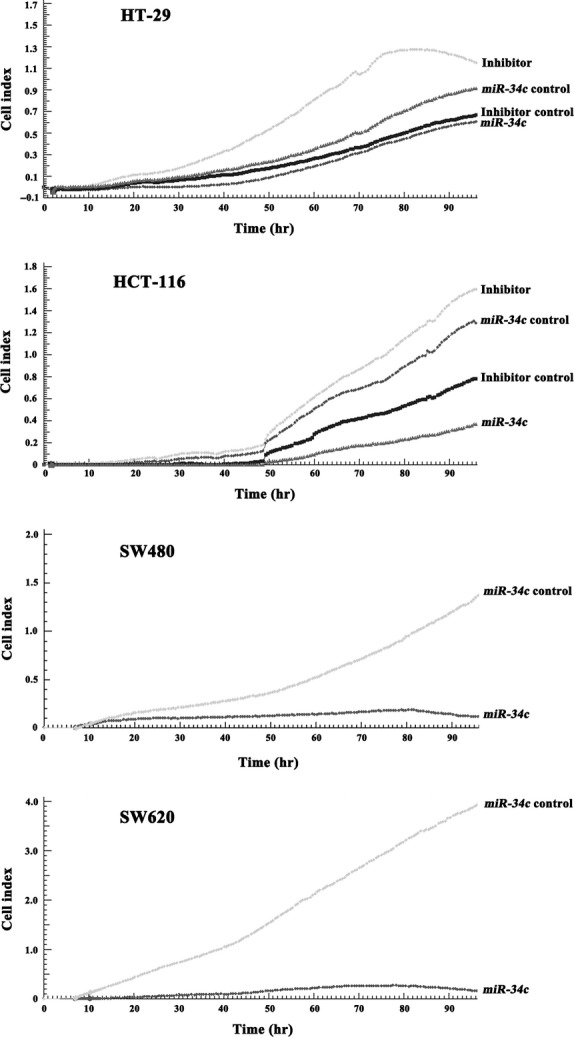
Real-time CRC cell growth was monitored for 96 hrs in cells infected with either *miR-34c* or its inhibitor. The cell index corresponds to cell proliferation. Infection with *miR-34c* apparently inhibited cell growth, while infection with the miR-34c inhibitor reversed the decrease.

**Figure 4:**
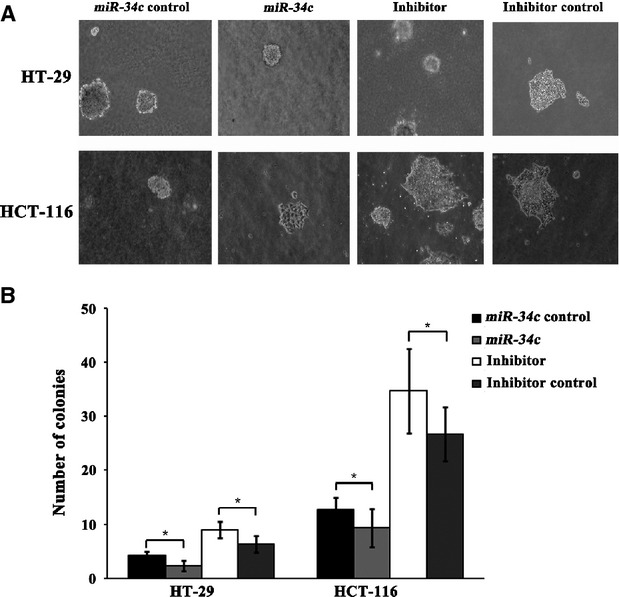
Soft agar colony formation assay. (**A**) Representative pictures of the colonies. (**B**) The number of colonies formed in ten randomly sampled visual fields (*P < 0.05)

**Figure 5:**
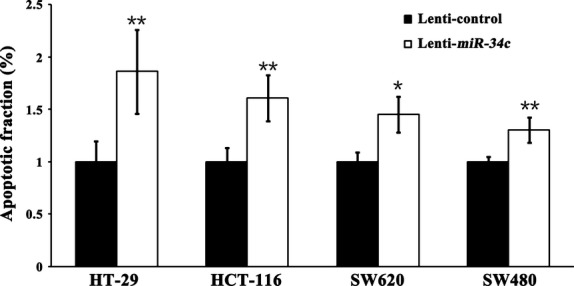
Overexpression of *miR-34c* significantly promoted apoptosis in CRC cells compared to the controls (*P < 0.05; **P < 0.01)

Next, we investigated the consequences of ectopic *miR-34c* expression on cellular proliferation and apoptosis. Overexpression of *miR-34c* resulted in a decrease in cellular proliferation by approximately 32.3% in HT-29 cells (*P* < 0.01), 71.1% in HCT-116 cells (*P* < 0.01), 91.5% in SW480 cells (*P* < 0.01) and 93.2% in SW620 cells (*P* < 0.01); on the other hand, silencing of *miR-34c* increased cell proliferation by 66.7% in HT-29 cells (*P* < 0.01) and 50.3% in HCT-116 cells (*P* < 0.01; Fig.3). Meanwhile, to determine whether the *miR-34c*-mediated down-regulation of KITLG could affect the capacity of CRC cells to grow in a semisolid medium, HT-29 and HCT-116 cells were seeded in soft agar and allowed to grow for 14 days, and the number of colonies from the HT-29 and HCT-116 cells infected with *miR-34c* was significantly less than those of the controls, while the number of colonies from cells infected with the *miR-34c* inhibitor was significantly increased compared to the controls (Fig.4). Moreover, infection with *miR-34c* induced more pronounced apoptosis, as determined by Annexin V/PI double-staining by using flow cytometry. The apoptotic fraction was elevated by 86.0% in HT-29 cells (*P* < 0.01), 60.5% in HCT-116 cells (*P* < 0.01), 45.2% in SW620 cells (*P* < 0.05) and 29.9% in SW480 cells (*P* < 0.05; Fig.5). These results support an inhibitory effect of *miR-34c* on CRC tumorigenesis, with variable extents in the different CRC cell lines.

### Disruption of *KITLG* by miR-*34c* abrogates cellular migration and invasion

**Figure 6:**
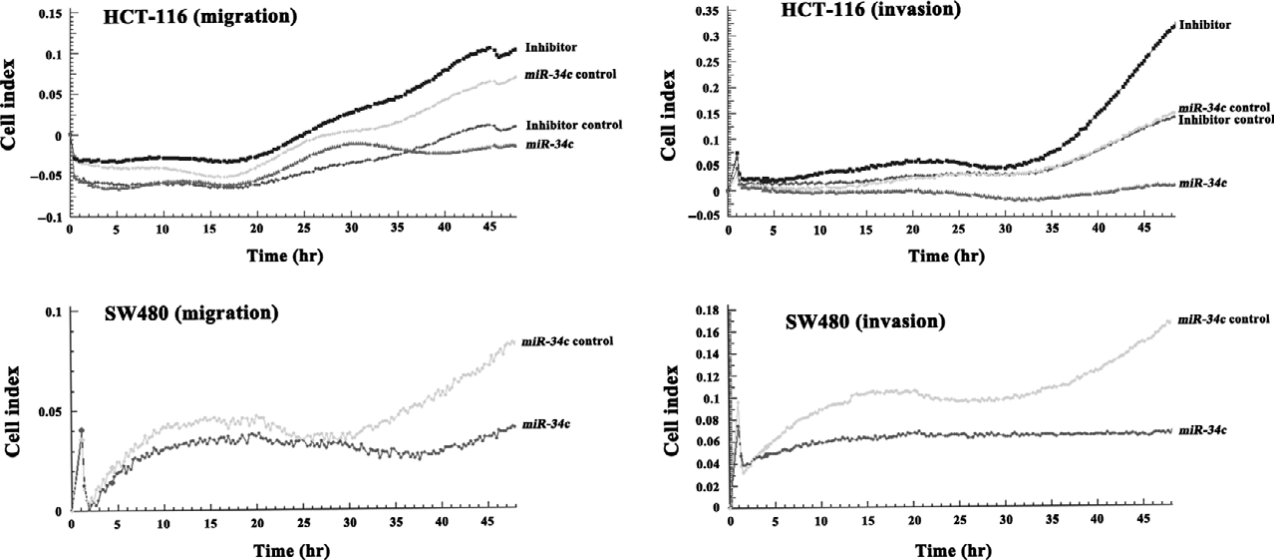
Effect of *miR-34c* on the migratory and invasive capacities of the HCT-116 and SW480 cells.

It has been reported that exogenous *KITLG* enhanced the cellular migration and invasion of the Colo320 CRC cell line 29. Here, we tested whether infection with *miR-34c* might interfere with the migratory and invasive capacities of HCT-116 and SW480 cells. As determined by RTCA, the ectopic expression of *miR-34c* led to a significant reduction in the migratory capacity of HCT-116 (52.0%, *P* < 0.01) and SW480 cells (70.0%, *P* < 0.01; Fig.6). The invasive capacity of the cells to migrate across a Matrigel biofilm was decreased by 77.8% (*P* < 0.01) in SW480 cells and 95.7% in HCT-116 cells (*P* < 0.01) after *miR-34c* infection. Conversely, the silencing of *miR-34c* enhanced the migratory (50.0%, *P* < 0.05) and invasive (80.8%, *P* < 0.01) abilities of HCT-116 cells (Fig.6).

### Knockdown of KITLG by specific siRNA contributes to tumour suppression

**Figure 7:**
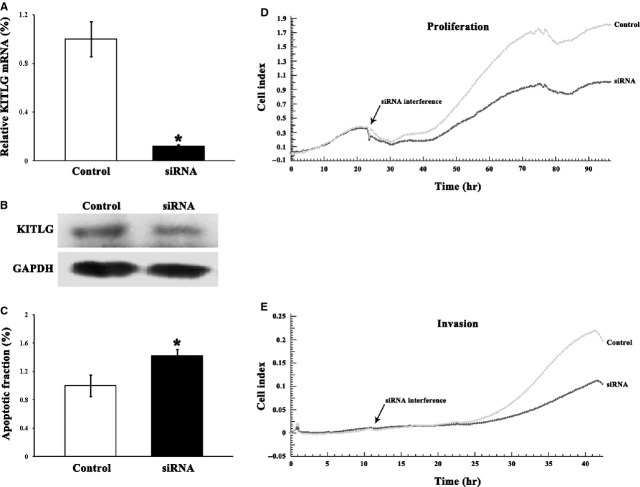
Forty-eight hours after transfection, siRNA efficiently knocked down KITLG at mRNA (**A**) and protein (**B**) levels in HCT-116 cells. (**C**) siRNA against KITLG significantly promoted apoptosis. (**D**) Cellular proliferation was dramatically decreased in cells transfected with KITLG-specific siRNA. (**E**) The invasive capacity of the HCT-116 cells was markedly inhibited after KITLG-specific siRNA transfection.

To observe that the tumour-suppressive effects of *miR-34c* are caused by regulation of KITLG, KITLG was effectively knocked down by 88.1% in HCT-116 cells by its specific siRNA (Fig.7). The down-regulation of KITLG suppressed cellular proliferation by 31.8%, invasion by 46.1%, and increased apoptosis by 42.2%, respectively (Fig.7).

## Discussion

A growing number of studies have shown that the overexpression of KITLG in CRC cells is correlated with tumorigenesis and metastasis 25,27,29, which prompted us to examine the regulation of KITLG expression in CRC cells. MicroRNAs are one of the important post-transcriptional regulators of their target genes. In the current study, we provided evidence that *KITLG* is a new target of *miR-34c*, and *miR-34c* activation negatively regulates KITLG-mediated cellular proliferation, transformation, apoptosis, migration and invasion of CRC cells.

KITLG is absent in normal colonic epithelium, but it has been detected in most cancer specimens 27. In contrast, *miR-34c* is hypo-expressed in CRC tumour tissues because of CpG methylation of its promoter region 7,14,30. In the present study, we showed that KITLG was detected in HT-29, HCT-116, SW480 and SW620 cells, and the level of *KITLG* mRNA was reversely correlated with the level of *miR-34c*. *In silico* analysis predicted that the *KITLG* 3′UTR contains *miR-34c* binding sites, and a dual-luciferase reporter assay further confirmed that *miR-34c* represses luciferase activity by targeting the binding sites in the *KITLG* 3′UTR, indicating a direct interaction between *miR-34c* and *KITLG* mRNA. MicroRNAs control their target mRNAs through degradation and/or translational repression. Kaller *et al*. showed that one member of the *miR-34* family, *miR-34a*, mediates its major target mRNAs through both degradation and translational repression 31. In line with Kaller *et al*. 31, Siemens *et al*. 32 suggested that the up-regulation of *miR-34a* is sufficient to reduce the expression of its target, c-KIT, at the mRNA and protein levels 32. Here, we demonstrated that the ectopic expression of another member of the *miR-34* family, *miR-34c*, caused a reduction in *KITLG* mRNA and protein in CRC cells, suggesting that *miR-34c* could affect both *KITLG* mRNA degradation and translational repression. The *miR-34c*-mediated down-regulation of KITLG protein could be counteracted by the introduction of a *miR-34c* specific inhibitor. Collectively, our results suggested that *miR-34c* critically regulates the expression of KITLG in CRC cells.

Several lines of evidence have established that KITLG and its tyrosine kinase receptor, c-KIT, are involved in tumorigenesis. One general mechanism of c-KIT activation in malignant cells has been described as an autocrine/paracrine stimulation loop triggered by its ligand KITLG 25. Tumorigenesis results from a disruption of the balance between proliferation and apoptosis. Krasagakis *et al*. found that the proliferation index is in generally high and the apoptosis rate is low in Merkel cell carcinoma, which co-expresses c-KIT and KITLG 33. The c-KIT/KITLG axis plays an important role in the self-renewal and proliferation of cancer stem cells in non-small-cell lung cancer 22. In this study, we showed that KITLG expression was significantly suppressed after infection with *miR-34c*, and the *miR-34c*-induced down-regulation of KITLG inhibited proliferation but promoted apoptosis in CRC cells that concomitantly express c-KIT and KITLG. To evaluate the potential role of *miR-34c* in affecting anchorage-independent growth, which is an important malignant property of tumour cells, the four CRC cell lines were infected with *miR-34c* or its inhibitor; these experiments showed that *miR-34c* could inhibit the growth of tumour cell colonies. Moreover, our results demonstrated that the migratory and invasive abilities of the HCT-116 and SW480 cells were significantly suppressed following infection with *miR-34c*. We performed an additional *in silico* analysis *via* the cBio Cancer Genomics Portal (http://cbioportal.org). There are 59 invasive colorectal adenocarcinoma cases available in the database; among them, 1 case (TCGA-AA-3680) shows homozygous deletion of *miR-34c* and 3 cases (TCGA-AA-3561, TCGA-AG-3586 and TCGA-AG-3892) exhibit *KIT* mRNA up-regulation but none cases show decreased *miR-34a* or KITLG alteration. These *in silico* data along with the results from Siemens *et al*. 32 and our present study suggest that *miR-34* and KIT/KITLG signalling might be involved in the invasive CRC, which still needs further experiment to confirm. Furthermore, to show a critical role of KITLG down-regulation for the tumour-suppressive effects of *miR-34c* in CRC cells, siRNA-mediated knockdown of KITLG was applied. siRNA against KITLG in HCT-116 cells resulted in significant inhibition in cellular proliferation and invasion, but promotion in apoptosis. Thus, we proposed that *miR-34c* might function as a tumour suppressor in CRC by targeting KITLG to disrupt the c-KIT/KITLG autocrine/paracrine stimulation loop. It was noted that the outcome of KITLG knockdown was not completely parallel with what *miR-34c* overexpression brought about, indicating that there are likely other targets of *miR-34c* implicated in the CRC suppression.

The *miR-34* family is composed of *miR-34a*,*miR-34b* and *miR-34c* and mediates tumour cell activities through cell cycle arrest, apoptosis or senescence, and metastasis 34–36, depending on its targets, such as the oncogenes *BCL-2*,*c-MYC* and *c-MET* 8,10,35. Recently, Siemens *et al*. demonstrated that the *miR-34* family, particularly *miR-34a*, represses c-KIT in CRC cell lines, which interferes with several c-KIT-mediated effects in CRC cells 32. They further found that, after stimulation with KITLG, Colo320 CRC cells displayed increased migration and invasion, whereas ectopic expression of *miR-34a* inhibited the KITLG-induced migration and invasion 32. They concluded that the tumour-suppressive role of *miR-34a* may be potentially relevant to the repressed c-KIT expression in CRC cells 32. We found that both *miR-34a* and *miR-34c* could repress the expressions of c-KIT and KITLG by the use of *in silico* analysis and dual-luciferase reporter assays; and *miR-34a* more significantly down-regulated c-KIT expression than *miR-34c* did, while *miR-34c* played a more robust role in down-regulating KITLG expression than *miR-34a* did (data not shown). It seems that *miR-34a* mainly down-regulated the c-KIT expression, while *miR-34c* mainly down-regulated the KITLG expression; and the mechanism is needed to be studied in the future. Since Siemens *et al*. 32 have reported the regulation of c-KIT by *miR-34* family, we here only reported the regulation of KITLG by *miR-34c* which showed a more significant suppression of KITLG expression to avoid the re-publication of similar data. Suppressed KITLG expression by *miR-34c* in CRC cell lines consequently inhibited cellular transformation, proliferation, migration and invasion, as well as promoted apoptosis. Clinical observations revealed that the aberrant expression of the c-KIT/KITLG system is involved in a subgroup of CRC. Although only a small proportion of CRC patients expressed c-KIT in their tumours 37,38, the concomitant expression of c-KIT and KITLG is significantly associated with worse clinical outcome 27, most likely caused by the high proliferation rate and the low apoptosis rate of these tumour cells. Given that proliferation and apoptosis, as well as invasion, are critically correlated with the c-KIT/KITLG system, we propose that *miR-34a/c* might suppress these activities in CRC cells by inhibiting the expression of ' and KITLG.

Activation of c-KIT by KITLG results in the activation of several downstream cascades, such as the MAPK, STAT and PI3K/Akt pathways. The KITLG-enhanced proliferation and invasion of KIT-positive CRC cells has been shown to be achieved mainly through the PI3K/Akt pathway 29. Interestingly, *miR-34c* also directly down-regulates the expression of c-MET, which causes similar alterations in its downstream target, p-Akt 39. Collectively, the PI3K/Akt pathway might be influenced by the *miR-34c*-mediated down-regulation of KITLG expression in CRC, and this possibility should be investigated. Moreover, the *miR-34* family is involved in Wnt signalling cascades by suppressing the transcriptional activity of the β-catenin-T cell factor/lymphoid enhancer factor (LEF) complexes by targeting a set of Wnt pathway-regulated genes, including WNT1, WNT3, LRP6, β-catenin, LEF1 and Axin2. We also noticed that there was cross-talk between the PI3K/Akt and Wnt pathways *via* GSK-3β, which can possibly expand and enhance the inhibitory effects of *miR-34* in cancers 11,12.

In conclusion, our present study demonstrated the tumour-\suppressive abilities of *miR-34c* in CRC cells by targeting KITLG *in vitro*. Restoration of *miR-34c* expression resulted in the inhibition of cell growth, transformation, migration and invasion, accompanied by decreased KITLG expression. The *miR-34c*-KITLG pathway may be helpful in enhancing our understanding of the molecular mechanisms of CRC tumorigenesis and metastasis. Taken together, our findings suggested that *miR-34c* likely has a diagnostic advantage and may be a novel molecular target for CRC patients.

## Supporting Information

Figure S1 The vectors used in this study, including GV306, GV280, GV262 and GV217 from Genechem, Shanghai.

Figure S2 Immunofluorescence staining (upper panels) showed that HT-29 cells were labelled with KITLG (red), and most of the cells were infected with the lentivirus expressing EGFP (green). Real-time PCR (lower panel) confirmed that *miR-34c* expression was markedly increased in the HT-29 cells after infection with lenti-*miR-34c* (***P* < 0.01), whereas the expression of the unrelated *miR-214* did not affect the levels of *miR-34c* (*P* > 0.05). Similar results from the HCT-116, SW480 and SW620 cells are not shown here.
