# Supplementary figures and images for: KITLG is a novel target of miR-34c that is associated with the inhibition of growth and invasion in colorectal cancer cells
Source: J Cell Mol Med. 2014 Sep 12;18(10):2092–102. doi: 10.1111/jcmm.12368 (PMC4244023; doi:10.1111/jcmm.12368)

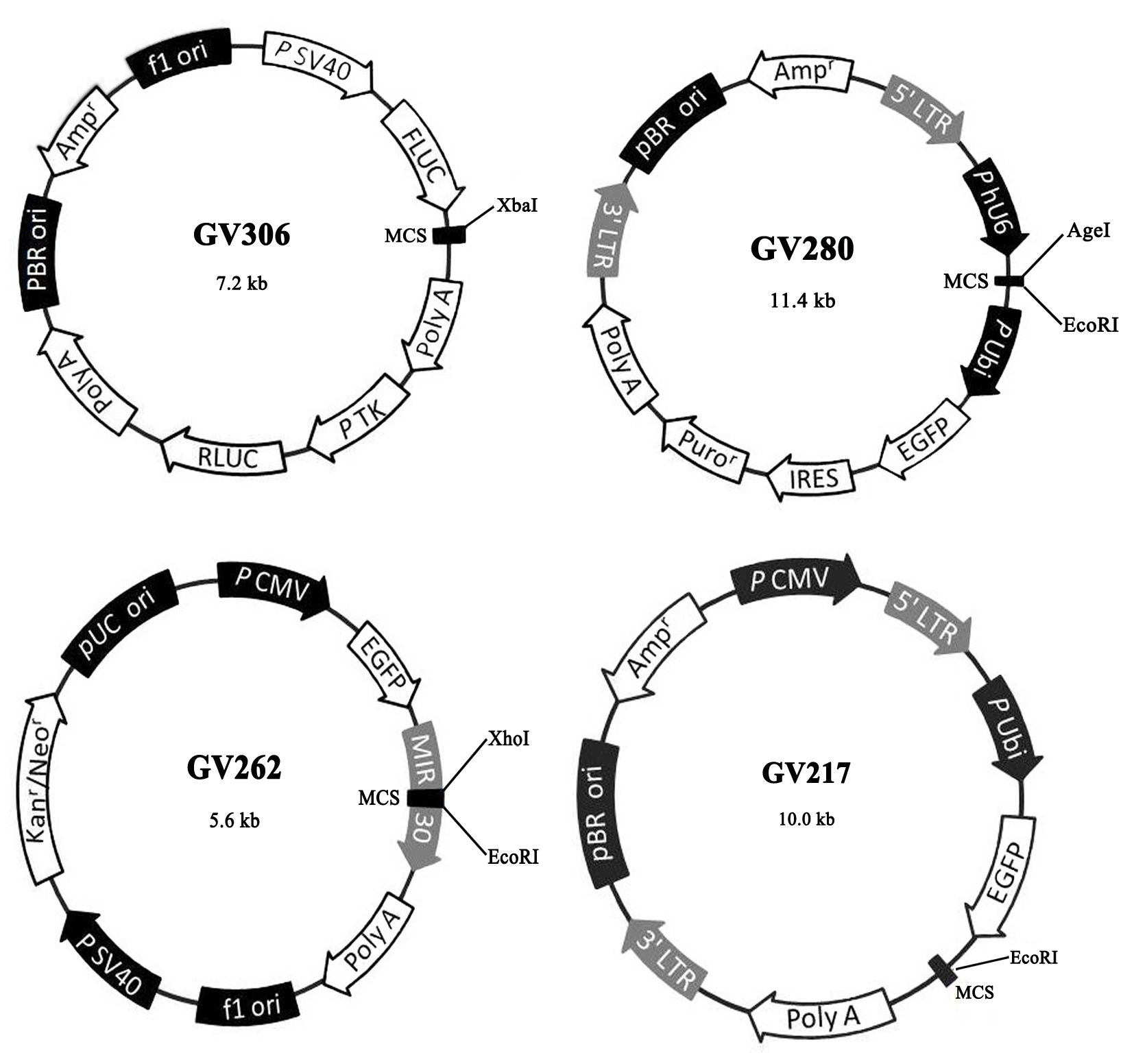

Supplement: Figure S1 — The vectors used in this study, including GV306, GV280, GV262 and GV217 from Genechem, Shanghai. [file jcmm0018-2092-sd1.tif]

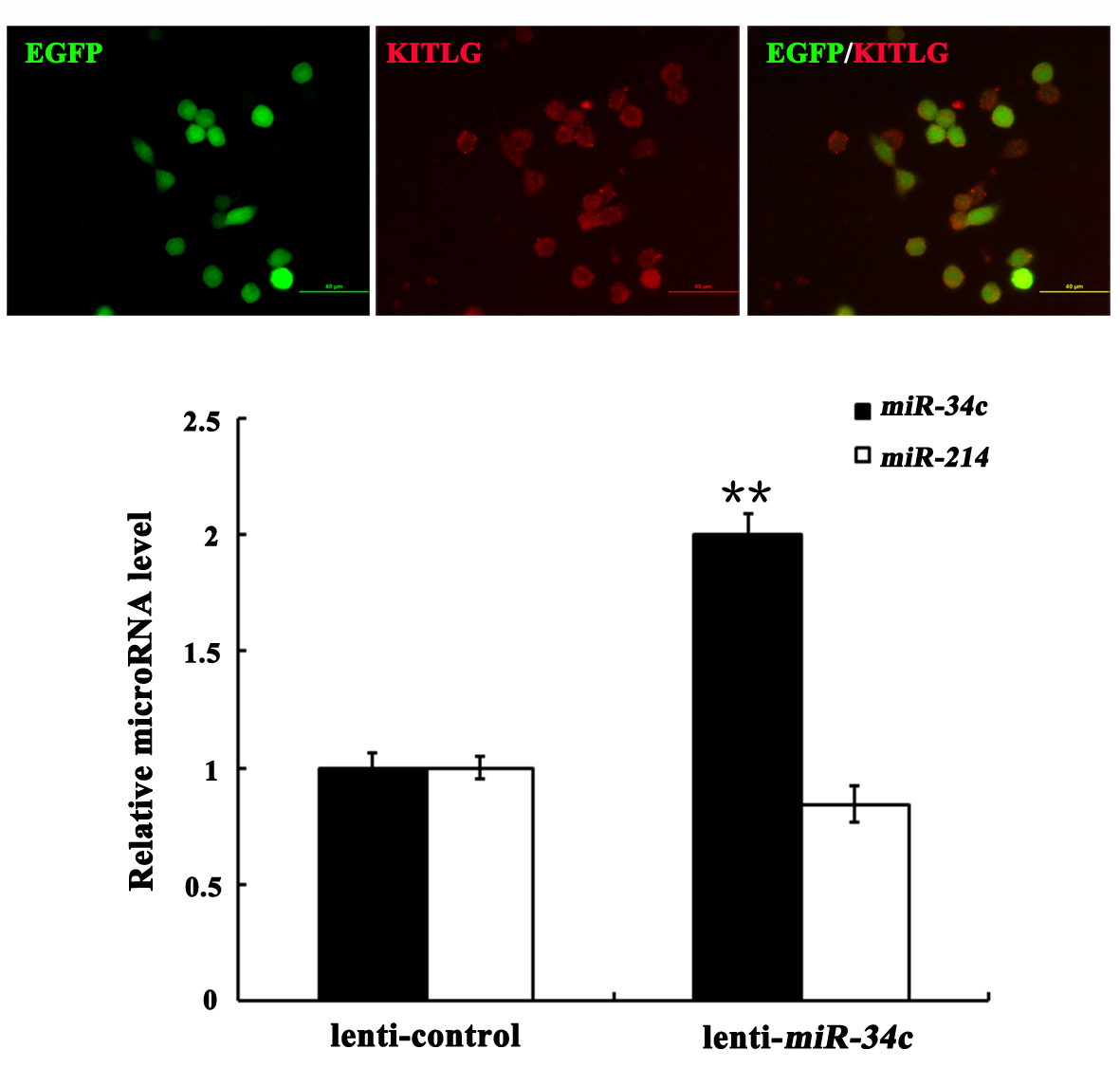

Supplement: Figure S2 — Immunofluorescence staining (upper panels) showed that HT-29 cells were labelled with KITLG (red), and most of the cells were infected with the lentivirus expressing EGFP (green). Real-time PCR (lower panel) confirmed that miR-34c expression was markedly increased in the HT-29 cells after infection with lenti-miR-34c (**P < 0.01), whereas the expression of the unrelated miR-214 did not affect the levels of miR-34c (P > 0.05). Similar results from the HCT-116, SW480 and SW620 cells are not shown here. [file jcmm0018-2092-sd2.tif]
